# The course of bipolar disorder in pregnant versus non-pregnant women

**DOI:** 10.1186/s40345-021-00239-z

**Published:** 2021-11-04

**Authors:** Anja W. M. M. Stevens, Stasja Draisma, Peter J. J. Goossens, Birit F. P. Broekman, Adriaan Honig, Elise A. M. Knoppert-van der Klein, Willem A. Nolen, Robert M. Post, R. W. Kupka

**Affiliations:** 1 Center for Bipolar Disorders, Dimence Mental Health, Deventer, The Netherlands; 2 Amsterdam UMC, Vrije Universiteit, Psychiatry, Amsterdam Public Health Research Institute, Amsterdam, The Netherlands; 3 GGZinGeest Specialized Mental Health Care, Research and Innovation Amsterdam, Amsterdam, The Netherlands; 4 Department of Public Health, Faculty of Medicine and Health Sciences, University Centre for Nursing and Midwifery, Ghent University, Ghent, Belgium; 5 Department of Psychiatry, OLVG Hospital, Amsterdam, The Netherlands; 6 GGZ Rivierduinen Alphen a/d Rijn, Amsterdam, The Netherlands; 7 Department of Psychiatry, University Medical Center Groningen, University of Groningen, Groningen, The Netherlands; 8 Bipolar Collaborative Network, Bethesda, MD USA; 9 Department of Psychiatry and Behavioral Sciences, District of Columbia, George Washington University, Washington, USA

**Keywords:** Bipolar disorder, Course, Pregnancy, Life chart method

## Abstract

**Background and rationale:**

Although it has been suggested that pregnancy may influence the course of bipolar disorder (BD), studies show contradictory results. Until now, no studies included a finegrained validated method to report mood symptoms on a daily basis, such as the lifechart method (LCM). The aim of the present study is to investigate the course of BD during pregnancy by comparing LCM scores of pregnant and non-pregnant women.

**Methods:**

Study design: Comparison of LCM scores of two prospective observational BD cohort studies, a cohort of pregnant women (n = 34) and a cohort of non-pregnant women of childbearing age (n = 52). Main study parameters are: (1) proportions of symptomatic and non-symptomatic days; (2) symptom severity, frequency, and duration of episodes; (3) state sequences, longitudinal variation of symptom severity scores.

**Results:**

No differences in clinical course variables (symptomatic days, average severity scores, frequency, and duration of episodes in BD were found between pregnant and non-pregnant women. With a combination of State Sequence Analysis (SSA) and cluster analysis on the sequences of daily mood scores three comparable clusters were found in both samples: euthymic, moderately ill and severely ill. The distribution differences between pregnant and non-pregnant women were significant, with a majority of the pregnant women (68%) belonging to the moderately ill cluster and a majority of the non-pregnant women (46%) to the euthymic cluster. In pregnant women the average daily variation in mood symptoms as assessed with Shannon’s entropy was less than in non-pregnant women (respectively 0.43 versus 0.56).

**Conclusions:**

Although the use of daily mood scores revealed no difference in overall course of BD in pregnant versus non-pregnant women, more pregnant than non-pregnant women belonged to the moderately ill cluster, and during pregnancy the variation in mood state was less than in non-pregnant women. Further research is necessary to clarify these findings.

## Introduction and rationale

Bipolar disorder (BD) is a recurrent mental illness characterized by depressive, hypomanic, and/or manic episodes separated by euthymic intervals and usually manifests in young adulthood (Goodwin and Jamison 2007). The lifetime prevalence ranges from 1.3% to 2.4% (Merikangas et al. 2011; Graaf et al. 1996).

Patients with BD show considerable illness-related morbidity (Post et al. 2003; Ferrari et al. 2016) and the disorder significantly influences their wellbeing and social, occupational, and general functioning (Altshuler et al. 2006; Bonnin et al. 2012; Parker et al. 2018).

In clinical practice, women with BD often ask their physician about the impact of pregnancy on the course of their illness. However, research into the relation between pregnancy and course of BD is scarce. Unlike repeated findings that the postpartum period has a negative influence on the course of BD, with an overall risk of relapse of 35% (66% or medication-free women and 23% for women who used prophylactic medication) (Wesseloo et al. 2016), the impact of pregnancy itself on the course BD is still uncertain, and various studies have reported conflicting results (McNeil et al. 1984; Sharma and Persad 1995).

While some population-based studies suggest that pregnancy could be protective with low rates of new onset and relapse during this period (Munk-Olsen et al. 2006,2009), clinical studies provide conflicting findings. Most of the older studies are retrospective, and most of the prospective studies report high recurrence rates in women who discontinue mood stabilizers (Sharma and Pope 2012).

In a retrospective study (Viguera et al. 2011), clinical data were pooled of 2252 pregnancies of women with BD and unipolar depression. Rates of affective episodes and risk factors were identified during pregnancy and the postpartum period. Among women with BD, 23% had illness episodes during pregnancy, compared to 4.6% of women with unipolar depression. Risk factors were younger age at illness onset, previous postpartum episodes, shorter duration of illness, having fewer children, and not being married.

Freeman et al. (2002) interviewed 30 women with BD after pregnancy with a structured clinical interview, and found that 15 (50%) reported no change or fewer mood symptoms during pregnancy, while the other half reported more symptoms. The experience of worsening of mood symptoms during pregnancy also predicted postpartum recurrence (Freeman et al. 2002). A limitation of this study is that the assessment was retrospective and thus prone to recall bias.

Grof et al. found a protective effect of pregnancy on the frequency and duration of mood episodes in a sample of 28 women with BD (with 56 pregnancies), who had become pregnant prior to receiving successful lithium prophylactic treatment (Grof et al. 2000). Retrospectively, they compared illness severity during the 9 months of pregnancy with the 9 months before pregnancy intra-individually. The recurrence risk during pregnancy was markedly lower and recurrences of mood episodes were significantly shorter during pregnancy in comparison to pre-pregnancy.

In a recent review of the influence of pregnancy on the course of BD we concluded that despite the importance of the topic there is a paucity of evidence on recurrence rates of mood episodes during pregnancy among women with BD (Stevens et al. 2019). Another review also stated that the literature cannot answer the question of how pregnancy affects the course of BD, but merely informs us about the effect of discontinuation of medication in pregnancy (Salim et al. 2018). Retrospective studies are more sensitive to recall bias and results are therefore less reliable, while prospective studies focused mainly on the effect of discontinuation of medication and hardly on the relation between pregnancy and course of illness.

Moreover, none of these studies used a detailed mood monitoring method to assess course of illness during pregnancy. The LifeChart Method (LCM) (Leverich et al. 2001; Denicoff et al. 2000) is a prospective assessment of fluctuations and severity in mood on a daily basis, resulting in more precision, with less risk for recall bias as compared with retrospective self reported data that were collected in most studies (Draisma et al. 2015).

The aim of the present study is to prospectively investigate the relationship between pregnancy and the course of BD.

## Methods

LCM-data of two observational, prospective cohort studies were used to compare the course of BD in pregnant versus non-pregnant women.

### Study samples

The research samples were (1) pregnant Dutch women with an established DSM-IV diagnosis of BD, who participated in the Sleepreg-BD study between 2012 and 2018 (n = 34), and (2) non-pregnant Dutch women of childbearing age with an established DSM-IV diagnosis of BD, who participated in The Stanley Foundation Bipolar Treatment Outcome Network study between 1995 and 2000 (n = 52).

The Sleepreg-BD study was a multi-site study in the Netherlands investigating the effect of sleep disturbance in pregnancy and the perinatal period on postpartum psychopathology in Dutch women with BD (Stevens et al. 2014). Pregnant women, aged 18–45 year, with a diagnosis of BD were asked to fill in the LCM from week 13 of pregnancy till 12 weeks postpartum. To avoid including women whose pregnancy ended prematurely, we recruted pregnant women at the end of the first trimester of pregnancy. To investigate the illness course during pregnancy, for this study LCM postpartum scores were not included in the analysis. The LCM was completed by 46 women, of these 34 met the inclusion criterion of at least 60 days of LCM reports.

The Stanley Foundation Bipolar Network (SFBN) was a multi-site research program coordinated at the National Institute of Mental Health (NIMH), with four clinical centers in the USA, two in Germany and one in the Netherlands, as described in detail elsewhere (Leverich et al. 2001; Post et al. 2001). Its main aim was to evaluate the long-term illness course and define longitudinal illness patterns, and to investigate the effectiveness of conventional and novel pharmacological treatments in a large group of patients with BD. The SFBN database consisted of over 900 patients, including n = 174 from the Netherlands. For this current study we included data of the 52 non-pregnant women, age 18–45 year, and included in the Netherlands, who had completed at least one full year of LCM. Since the pregnant women delivered a maximum of 272 daily severity scores, we selected the 272 completed last lifechart ratings from the first year of prospective follow-up of the SFBN women for comparison with the Dutch women of the Sleepreg-BD study.

### Assessment instruments and outcome variables

#### Patient and clinician questionnaires:

The Stanley Foundation Bipolar Treatment Outcome Network designed two questionnaires to collect basic demographic and clinical data from both patients and clinicians. These questionnaires (Questionnaire for Bipolar disorder, QBP) generate a comprehensive overview of the characteristics of participants as well as illness and treatment history (Leverich et al. 2001; Suppes et al. 2001), and were translated into Dutch.

QBP reports of medication use were transformed into categories (0: no medication; 1: one mood stabilizer; 2: more than one mood stabilizer; 3: mood stabilizer and other psychotropic medications; 4: only other psychotropic medications).

#### LCM

The LCM provides a graphic representation of minor mood swings and major mood episodes, and can be used both retrospectively and prospectively (Denicoff et al. 2000,1997). The impact of daily mood symptoms on functioning is rated on a 5-point scale (0 = no dysfunction or euthymia, 1 = mild, 2 = low moderate, 3 = high moderate, 4 = severe dysfunction). Since severity is rated for two poles: mania and depression, this results in nine different ratings. Patients of both cohort studies were asked to complete a prospective LCM on a daily basis during the entire study period. Illness course variables could be calculated, thus allowing longitudinal assessment of illness patterns (Born et al. 2009; Kupka et al. 2007; Nolen et al. 2004). Relevant variables were: number, duration, and severity of mood symptoms and mood episodes, and proportion of time ill during the observation period. A validation study reported high correlations between LCM ratings and ratings on the Young Mania Rating Scale (YMRS) (*r* = 0.656*, p* < 0.001) and the Inventory of Depressive Symptomatology-Clinician (IDS-C) (*r* = 0.875, *p* < 0.001) (Denicoff et al. 2000). Draisma et al. found a Spearman’s Rho of 0.61 between LCM depression scores and Clinical Global Impression (CGI-BD) rated depression, and a Rho of 0.63 between LCM mania scores and CGI-C rated mania in a sample of Dutch patients with BD (n = 137), denoting a strong association between LCM ratings and CGI scores (Draisma et al. 2015).

Thus, main study parameters where threefold: (1) demographical variables at baseline: age, marital status, educational level and work status at baseline; (2) clinical variables at baseline consisting of diagnosis, illness duration, age of onset, number of lifetime manic and depressive episodes, number of hospitalizations, use of medication, lifetime alcohol/drugs abuse, number of serious suicide attempts; and (3) LCM derived clinical variables such as proportion of time ill or impaired, number of days with scores not equal to five (i.e. the euthymic state), average illness severity scores, average duration of episodes, and frequency of episodes.

### Statistical analysis

Analyses were done in four steps: (1) demographic and clinical characteristics of the pregnant and non-pregnant samples were compared with descriptive statistics; (2) complete series of illness states – the LCM mood scores – were analyzed and typified for the two samples with the use of state sequence analysis (SSA); (3) cluster analysis was performed on the state sequences; and (4) two regression analyses were done, one on clusters with a set of predictors and another on variation in daily states with the same set of predictors.

The set of predictors were illness duration, marital status, work status, educational level, use of medication (Viguera et al. 2011; Akdeniz et al. 2003).

Differences in demographical and clinical variables were analyzed with descriptive statistics, using t-tests for continuous variables with normal distribution, Mann-Whitey tests for non-normal continuous variables and chi square tests for categorical variables. Descriptive analyses were performed in SPSS version 27. Missing lifechart scores of the 34 pregnant women within the observation period (less than 0.3% of the data) were imputed through intrapolation of the surrounding daily scores.

Complete series of illness states as expressed in fluctuations of daily severity scores of the pregnant and non-pregnant women were analysed with SSA (Roux et al. 2019; Gabadinho et al. 2011). The time series of a maximum of 272 daily severity scores resulted in so-called sequences of successive states. The goal of SSA is to describe complete sequences of events as trajectories of subjects though possible states. It is a non-parametric approach with no assumptions regarding underlying processes and aimed at description of sequences as a whole (Courgeau 2018).

Dissimilarities between state sequences were calculated with a dissimilarity measure based on the number of operations necessary to translate a specific sequence into another. The similarities and differences were based on optimal matching techniques. Subsequently, clustering methods were applied to build similar types of sequences (Abbott 1990). By clustering sequences, groups were formed that were as homogenous as possible within the group and as different as possible from other groups. In the analysis, hierarchical clustering was applied using Ward’s linkage on the distance matrix. The clustering procedures resulted in a typology of sequence courses. Sequences within a cluster had the lowest dissimilarity scores with each other, and between clusters dissimilarity was optimal. Distributions of women over the clusters found in the sample of pregnant women (n = 34) were subsequently compared to those found in the non-pregnant women (n = 52).

Shannon entropy, an indicator of diversity of states (~ severity scores) within sequences was calculated for all day. Entropy can vary between 0 (all sequences in the same state at time T), to 1 (maximum diversity at time T).

Multinomial regression of clusters on the predictors was performed. In the same vain regression of entropy on this set of covariates was applied.

The package TraMineR (version 2.2–0.1) within R-software was used for sequence analysis.

## Results

**Fig. 1:**
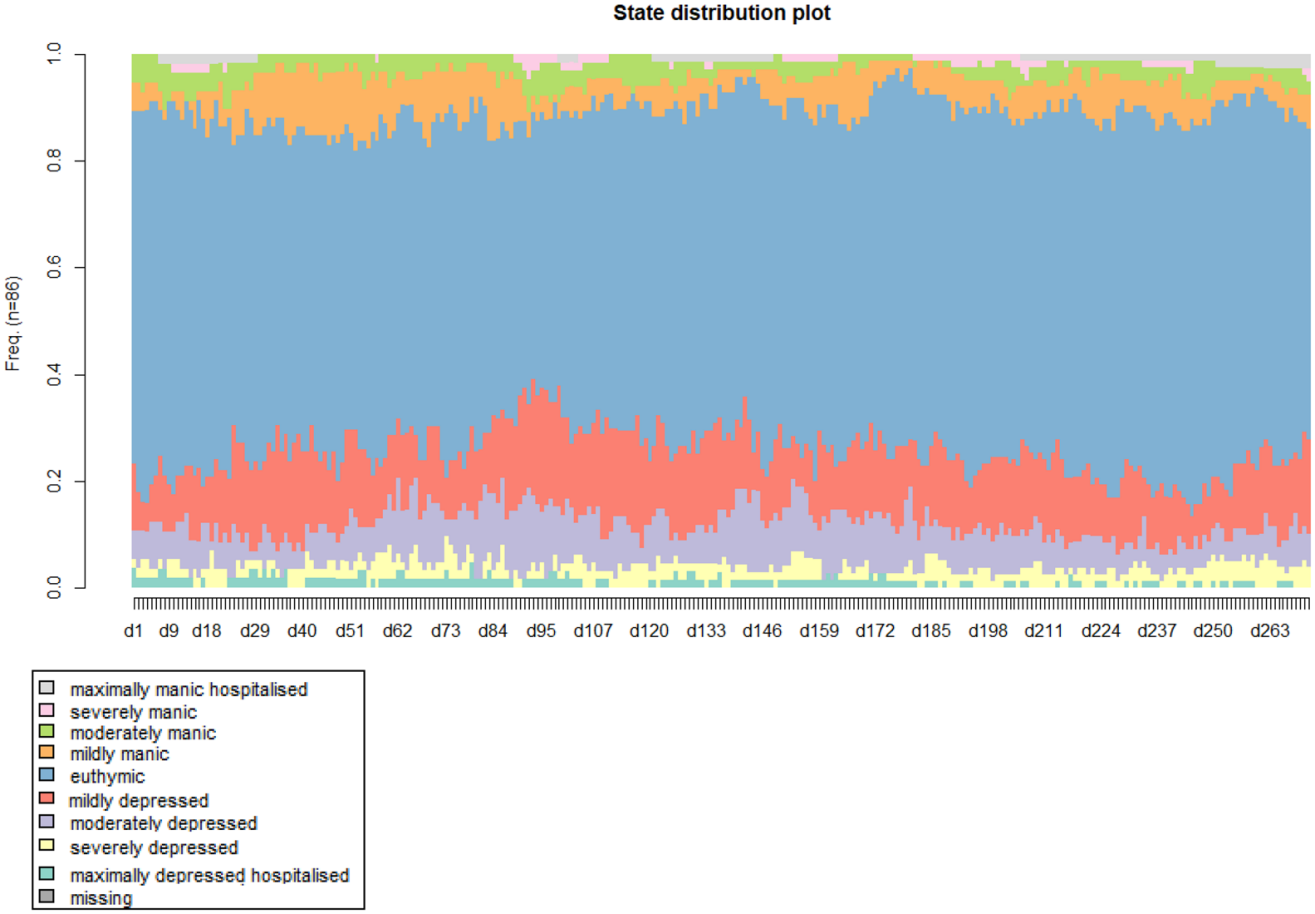
Index plot: distribution of states (daily self rated severity score) for both samples (pregnant and non-pregnant women, n = 86) over the observation period of 272 days. X-axis: time in days, Y axis: proportions women distributed over states described in the legend

**Fig. 2:**
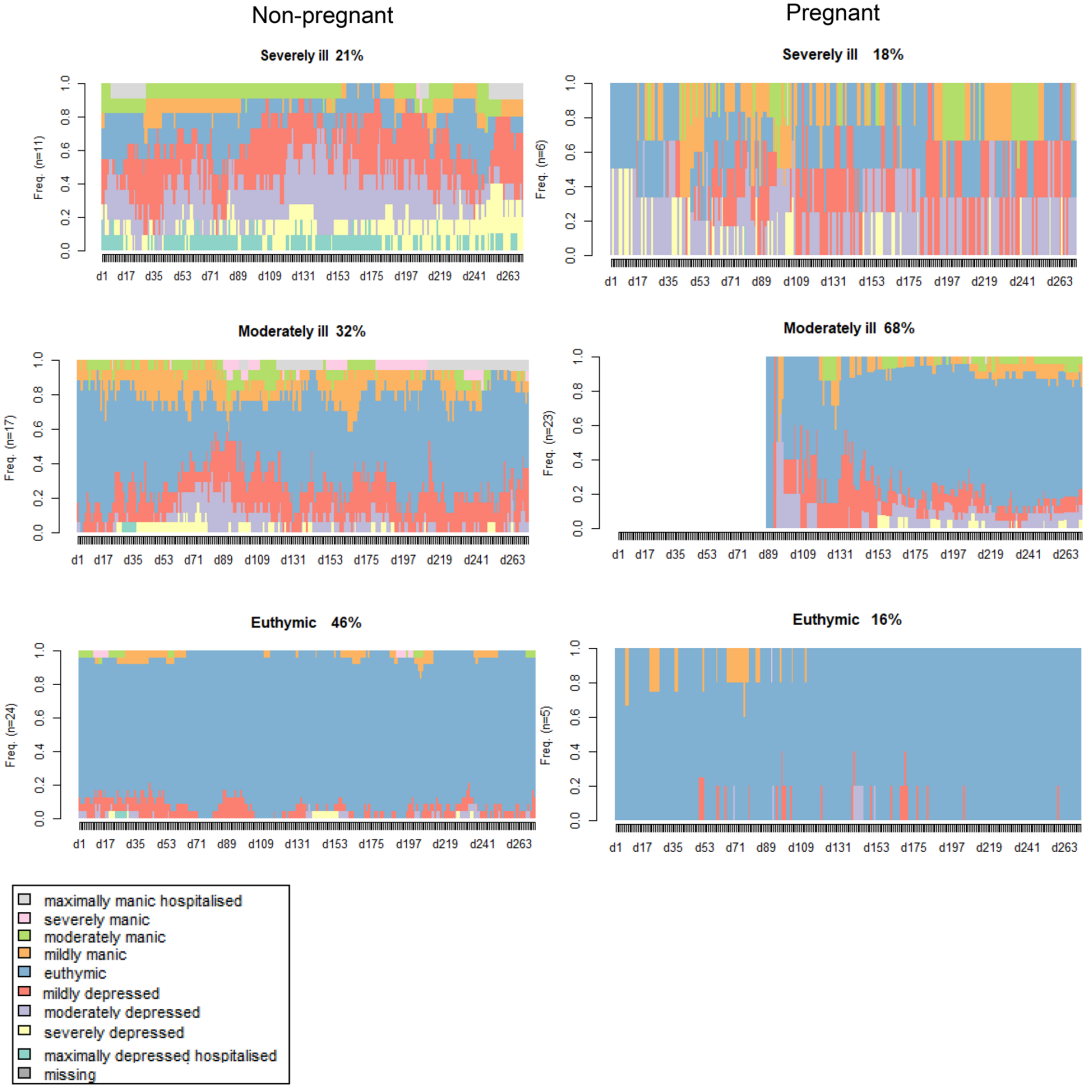
Index plots of three cluster solution for the pregnant sample and non-pregnant sample; X-axis: time in days (d1 means dey 1), Y axis: proportions women distributed over states in cluster

**Fig. 3:**
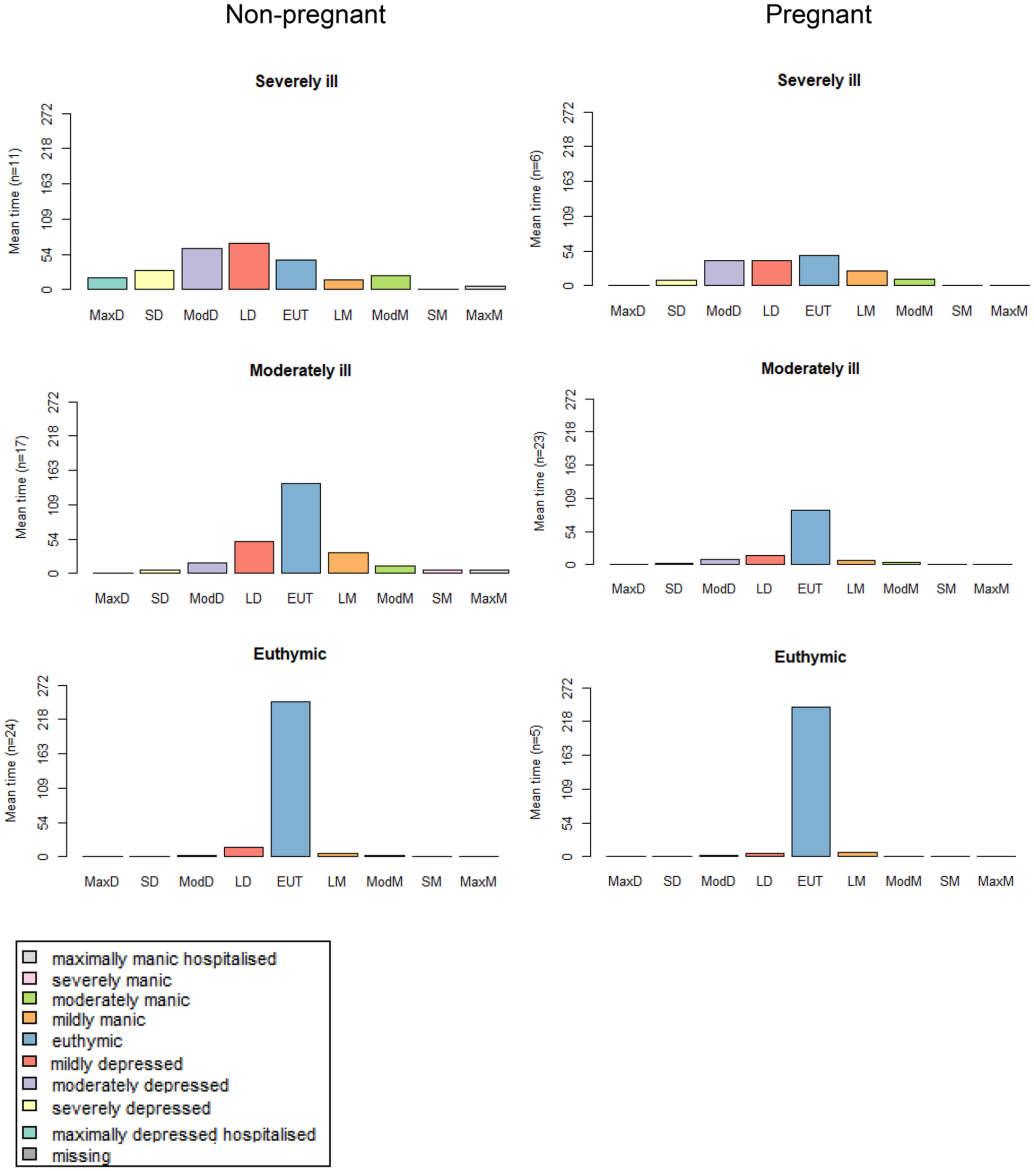
Mean time (in days) spent in each state for three clusters, pregnant women on the left, non-pregnant women on the right

First, descriptive statistics with respect to the comparisons of sample characteristics are presented in Tables 1, 2, 3. Next the distribution of states over days is presented in an index plot for all women in Fig. 1. Cluster solutions for both samples are also presented graphically as index plots in Fig. 2. Mean time spent in a cluster is given in Fig. 3. Results of multinomial regression of clusters in the combined set of samples on predictors are provided in Table 4. Finally, results of regression of entropy on the same set of predictors are presented.

**Table 1:** Demographic characteristics of non-pregnant and pregnant women with BD recruited from two study cohorts from the Netherlands

	Non-pregnant (n = 52)	Pregnant (n = 34)	Test statistic
Age^a^ Mean (SD)			
Years	35.2 (6.3)	34.1 (3.9)	T = -0.95 p = 0.35
Marital status n (%)			
Married/cohabitating	24 (46.2)	33 (97.1)	
Widowed/separated	3 (5.8)	0 (0)	
Single	25 (48.1)	1 (2.9)	χ^2^ = 23.2 p < 0.01
Educational level n (%)			
Low (some high school or less)	1 (1.9)	1 (3.0)	
Middle(high school-2 year college)	43 (82.7)	8 (24.2)	
High (graduate of professional school)	8 (15.4)	24 (72.7)	χ^2^ = 29.2 p < 0.01
Work n (%)			
Regular work, school or household	29 (59.2)	26 (78.8)	
Other work	19 (38.8)	2 (6.1)	
Unable to work	1 (2.0)	5 (15.2)	χ^2^ = 14.0 p < 0.01

^a^ Age was normally distributed (Kolmogorov_Smirnov > 0.05, Shapiro–Wilk > 0.05)

**Table 2:** Lifetime clinical characteristics of non-pregnant and pregnant women with BD recruited from two study cohorts from The Netherlands

Clinical characteristics	Non-pregnant (n-52)	Pregnant (n = 34)	Test statistic
Diagnosis, n (%)			
Bipolar I	38 (76.0)	19 (59.4)	
Bipolar II	12 (24.0)	13 (40.6)	χ^2^ = 2.541 p = 0.11
Illness duration^a^			
Mean duration in years (SD)	15.4 (7.29)	14.6 (6.18)	T = -5.02 p = 0.62
Less than 5 years	3 (5.8)	3 (9.1)	
5–9 years	9(17.3)	3 (9.1)	
10–19 years	21 (40.4)	17 (51.5)	
20–29 years	19 (36.5)	10 (30.3)	χ^2^ = 2.071 p = 0.56
Age of onset^a^ (mean, SD)	19.9 (5.93)	19.5 (5.23)	U = 828.5 p = 0.790
No. of depressive episodes, n (%)			
0 episodes	0 (0)	3 (9.1)	
1 episode	6 (11.5)	1 (3.0)	
2–4 episodes	16 (30.8)	16 (48.5)	
5–10 episodes	13 (25.0)	7 (21.2)	
11–20 episodes	5 (9.6)	4 (12.1)	
> 20 episodes	12 (23.1)	2 (6.1)	χ^2^ = 11.977 p = 0.04
No. of manic episodes, n (%)			
0	0	1 (3.0)	
1 episode	4 (7.7)	6 (18.2)	
2–4 episodes	20 (38.5)	19 (57.6)	
5–10 episodes	16 (30.8)	4 (12.1)	
11–20 episodes	7 (13.5)	1 (3.0)	
> 20 episodes	5 (9.6)	2 (6.1)	χ^2^ = 10.699 p = 0.06
Hospitalizations^a^, mean (SD)	4.1 (5.8)	1.4 (1.7)	
Medication, n (%)			
Without medication	0 (0.0)	8(23.5)	
1 Mood stabilizer	23 (44.2)	8 (23.5)	
> 1 Mood stablizers	20 (38.5)	0 (0.0)	
Moodstabilizer and other medication	9 (17.3)	8 (23.5)	
Other medication only	0 (0.0)	10 (29.4)	χ^2^ = 43.453 p < 0.01
Substance abuse/dependence, n (%)			
Alcohol	9 (17.3)	3 (9.1)	χ^2^ = 1.124 p = 0.29
Drugs	7 (13.5)	7 (21.5)	χ^2^ = 0.881 p = 0.35
Serious suicide attempts			
None	36 (69.2)	28 (84.8)	
One or more	16(30.8)	5 (15.2)	χ^2^ = 2.547 p = 0.10

^a^ Mean illness duration was normally distributed (Kolmogorov_Smirnov > 0.05, Shapiro–Wilk > 0.05), age of onset and number of hospitalizations were not (Kolmogorov_Smirnov < 0.05, Shapiro–Wilk < 0.05)

**Table 3:** Clinical lifechart results of non-pregnant and pregnant women with BD recruited from two study cohorts from The Netherlands

Averages (SD)^a^	Non-pregnant (n = 52)	Pregnant (n = 34)	Test statistic T	p
No. depressive episodes^a^	3.43 (11.95)	0.55 (0.94)	1.40	0.16
No. manic episodes^a^	0.35 (0.68)	0.38 (1.35)	− 0.14	0.89
No. hypomanic episodes^a^	1.10 (2.95)	1.29 (1.59)	− 0.40	0.69
No. manic days^a^	14.06 (36.53)	8.90 (20.81)	0.75	0.46
No. hypomanic days^a^	16.19 (23.12)	18.83 (33.25)	− 0.43	0.66
No. depressive days^a^	71.85 (76.07)	56.78 (80.20)	0.88	0.38
No. days ill (depressed, manic, hypomanic)^a^	102.10 (84.53)	84.51 (93.17)	0.91	0.37
Average mania score	0.39 (1.01)	0.23 (0.54)	0.85	0.40
Average depression score	0.45 (0.58)	0.34 (0.55)	0.88	0.38

^a^ Corrected for the number of observations

**Table 4:** Multinomial regression of clusters on a selection of predictors (n = 86)

	Odds ratio (Std. Error)	
	Cluster 2 (moderately ill)	Cluster 3 (euthymic)
Educational level	6.71 (0.67)**	1.64 (0.57)
Marital status	1.3e−06 (245.87)	1.2e−00 (0.48)
Work	1.59 (0.57)	1.14 (0.49)
Medication	1.06 (0.25)	0.87 (0.26)
Illness duration	0.75 (0.41)	1.29 (0.32)
Pseudo R^2^	0.19	

Odds ratio’s and standard errors. Base outcome (reference category) cluster 1: severely ill. **p < 0.01

No age differences were present between the samples. Pregnant women were significantly more often married, attained higher educational levels and were more often unable to work (Table 1). Pregnant women had less previous hospitalisations and used less medication than the non-pregnant group (Table 2). Of the pregnant women three women used antidepressant medication without a mood stabilizer, while in the non-pregnant group none of the women used an antidepressant without a mood stabiliser. No significant differences were found regarding lifetime illness duration, age of onset, lifetime number of experienced manic or depressive episodes, lifetime substance abuse or dependence, and number of attempted suicides.

Comparison of the course of BD in both samples showed no significant differences in LCM variables (Table 3) regarding episodes or number of ill days during the observation episode.

### Sequence analysis results

Distributions of proportions of severity scores per day were plotted for the whole observation periods in an index plot (Fig. 1).

Figure 1 shows that most time of women with BD was spent in the euthymic state. This state covered the largest surface per day during the whole observation period. The percentage of women in this state was the highest, followed by ‘mildly depressed’ and ‘mildly manic’.

Figure 2 gives the graphic results of a cluster analysis with three clusters for the separate samples.

Women in the clusters ‘severely ill’ of both samples showed sequences with mainly severe and moderate depressive or manic days and few euthymic states. The second set of clusters, ‘moderately ill’ contained state sequences with some illness, days with mild to moderately ill days with respect to both mania as well as depression. Finally, clusters ‘euthymic’ were typified by sequences with mainly euthymic days, the main area of these clusters were taken up by euthymic states. This three cluster solution shows that the severely ill cluster in the non-pregnant sample consisted of 21% (11/52) of the participants and in the pregnant sample 18% (6/34) of the participants. The euthymic cluster in the pregnant sample consisted of 16% (5/34) the women, whereas this euthymic cluster in the non-pregnant sample was larger, consisting of 46% (24/52) of the women. The majority of the pregnant women were clustered as ‘moderately ill’: the percentage in this cluster is twice as high as the percentage of non-pregnant women in this cluster (68% and 32%, respectively). These distribution differences between samples were significant: χ^2^ = 48.02, df = 2, p-value < 0.001.

Number of days spent in each of the nine states obviously differed over the three clusters. Evidently, more days were spent in the euthymic state in the euthymic cluster as compared to the other clusters. Interestingly, daily scores were not normally distributed over mania and depression. There was a tendency that more days were rated as depressed (left side of the cluster figures) than manic (right side of the figures) (Fig. 3).

The level of entropy of scores, i.e., the diversity of states on each day for both samples, for the non-pregnant women were higher (average 0.56) than for the pregnant women (average 0.43).

In multinomial regression the dependent variable (cluster) has three different categorical values. Regression of the three clusters on three demographic variables (educational level, marital status and work) and two clinical variables (medication, illness duration) showed only one significant relation, namely between educational level: the odds of going from cluster 1 (severely ill) to cluster 2 (moderately ill). Thus, the higher the level of education, the higher the odds of going from severely ill to moderately ill (Table 4).

Subsequently, the results of regression of entropy (n = 86) on the same set of predictors is shown (Table 5).

**Table 5:** Regressing Entropy on a selection of covariates (n = 86)

Variable	Beta	Std. error	*t* value	Pr. ( >|t|)
Intercept	0.594	0.214	2.779	0.007**
Educational level	− 0.134	0.054	− 2.481	0.016*
Marital status	− 0.002	0.053	− 0.039	0.969
Work	− 0.015	0.046	− 0.332	0.741
Medication	0.025	0.023	1.080	0.283
Illness duration	0.011	0.032	0.339	0.736

The only covariate that produced a significant effect in regression of entropy on predictors was educational level. This suggests that the lower the educational level, the more instability occurred in the course of BD.

## Discussion

While previous prospective studies have investigated risk of, and/or time to, recurrence of mood episodes during pregnancy in BD (Viguera et al. 2007; Bergink et al. 2012), this is the first study comparing fine-graded prospective illness course with the lifechart method (LCM) in pregnant and non-pregnant women with BD. No differences were found in illness severity variables, such as number of days ill, including days depressed, hypomanic, or manic, or average severity scores. However, with a cluster analysis of LCM data within both samples to reveal longitudinal illness patterns, within the study period, more pregnant women were moderately ill, whereas more non-pregnant women were euthymic.

In addition, pregnant women showed less variation of mood states than non-pregnant women. Women with a higher educational level in both samples were more likely to belong to a cluster moderately ill than to a cluster severely ill. An explanation for the relation between educational level and less mood instability could be that more adequate psycho-education is obtained by higher educated women. The higher educational level in the cluster moderately ill than in the cluster severely ill could be due to the fact that those women had a milder course of BD and therefore were able to get a higher education level.

Constructing typologies/groups, according to temporal data, of patients with BD has been done by other investigators. Post et al. determined the severity of illness in the first 258 outpatients in the SFBN who had 1 year of prospective LCM ratings (Post et al. 2003). Their typologies contain groups of patients who remained severely and almost continuously ill (26.4%), intermittently ill (40.7%), or minimally ill (32.9%) over the course of that year. The patterns of the ratings were visually assigned to the three groups by two independent investigators. Nowadays, with the application of state sequence analysis, clustering methods can be used to build similar types of illness course and so defining groups that are as homogenous as possible on the one hand and as different as possible from other groups on the other. This clustering method does not depend on subjective clinician ratings, but can be applied automatically using algorithms, enhancing reliability.

A possible explanation for the difference between our two samples, especially the higher proportion of euthymic days in non-pregnant women versus pregnant women, could be that more non-pregnant women used psychotropic medication during the observation period. The use of medication prevents recurrences in pregnant and non-pregnant women (Stevens et al. 2019; Larsen and Saric 2017; Kishi et al. 2020; Geddes and Miklowitz 2013). In more detail, in our study 23.5% of pregnant women used no medication at all, compared to none (0%) of the non-pregnant women, whereas 38.5% of the non-pregnant women used more than one mood stabilizer compared to none of the pregnant women. Although the use of antidepressant medication without a mood stabilizer is not recommended in bipolar disorder because of the risk of mood instability and switch to mania (Pacchiarotti et al. 2013), three pregnant women but none of the non-pregnant women used an antidepressant without a mood stabilizer.

All women in both samples received psychiatric treatment and most of the pregnant women had pre-pregnancy consultations. It is possible that women with a more severe BD, who would not stop medication because of fear for recurrence, decided not to attempt to conceive, which could explain why the number of medications used in the pregnant women in our study was relatively low.

In general, women with a childwish are recommended to be stabilized for a period of at least six months before becoming pregnant (Thomson and Sharma 2018). In a study of 70 women with BD, 45% who sought consultation for treatment options and risks during pregnancy had been advised to avoid pregnancy by a health care professional before consultation (Viguera et al. 2002). After the consultation 37% chose to avoid pregnancy with the most commonly reported reason being fears of adverse effect of medication on the development of the fetus (56%) or concerns of genetic transmission of BD to offspring (22%).

There still is a paucity of systematic data on the effects of pregnancy on the course of BD. In our study, with use of daily mood monitoring, no differences were found in the course of BD in pregnant versus non-pregnant women. Comparing trajectories may be a better way to study the effect of pregnancy on the course of BD. A major challenge would be to investigate the role of pregnancy on the ‘naturalistic’ (i.e. untreated) course of BD, since it is recommended to prescribe some form of preventive medication in patients with BD also during pregnancy (Yatham et al. 2018).

Our study has several strengths. It is the first study comparing prospective daily mood ratings in a sample of pregnant and non-pregnant women with BD. The samples did not differ in demographic characteristics such as nationality and age, nor in clinical variables such as type of diagnosis (BD I or II), illness duration, age of onset, lifetime number of manic and depressive episodes, lifetime substance abuse, and serious suicide attempts. Also, this is the first study using SSA and clustering methods on LCM data to reveal different clusters of illness course.

However, there are also several limitations. Since the design consists of two cohorts from different studies, and cases were not assigned at random or via case control procedures, the comparability of both samples remains uncertain. The pregnant women had a higher educational level and were more often married than the non-pregnant women. The use of medication differed between the two cohorts, with a potential impact on the course of BD. Also, LCM data from pregnant women were reported from week 12 of pregnancy untill giving birth, hence data on the first trimester are not included. An obvious limitation of this study is the relatively small sample sizes of pregnant and non-pregnant women. Selection bias cannot be ruled out completely: the fact that pregnant women more often belonged to the moderately ill cluster and non-pregnant women to the euthymic cluster may be due to the possibility that pregnant moderately ill women were more likely to participate, or that euthymic non-pregnant women were more inclined to enter such a study than moderately ill non-pregnant women. Randomization in a study with regard to a comparison of pregnant and non-pregnant women with BD however is not feasible. Obviously more research with larger sample sizes about the effect of pregnancy on the course of BD is required. However, the fact that these small samples did not differ with respect to descriptive clinical course variables, such as number of manic and number of ill days, yet did show clear differences in illness pattern during the observation period adds further strength to the approach of sequential analysis of daily mood monitoring in BD, even with small sample sizes.

## Conclusion

No differences in average values of clinical course variables in BD were found among pregnant women compared to non-pregnant women. The application of SSA to reveal patterns in the overall course in the observational period did show differences in proportions of pregnant versus non-pregnant women distributed over three clusters of sequences. More pregnant women showed a moderately ill pattern of daily mood scores whereas more non-pregnant women showed a euthymic pattern. One explanation is that more non-pregnant women used (more) psychotropic medications than pregnant women, which may have a protective effect. Pregnant women showed less variation in mood than non-pregnant women.

To answer the question whether pregnancy influences the course of BD, ideally, we need large, prospective case–control studies comparing pregnant and non-pregnant women. Another option is to compare within individuals the course of BD during pregnancy and during the year before.

## Data Availability

The datasets used and/or analysed during the current study are available from the corresponding author on reasonable request.

## References

[CR1] Abbott AH (1990). Measuring Resemblance in Sequence Data: An Optimal Matching Analysis of Musicians' Careers. Am J Sociol.

[CR2] Akdeniz F, Vahip S, Pirildar S, Vahip I, Doganer I, Bulut I (2003). Risk factors associated with childbearing-related episodes in women with bipolar disorder. Psychopathology.

[CR3] Altshuler LL, Post RM, Black DO, Keck PE, Nolen WA, Frye MA (2006). Subsyndromal depressive symptoms are associated with functional impairment in patients with bipolar disorder: results of a large, multisite study. J Clin Psychiatry.

[CR4] Bergink V, Bouvy PF, Vervoort JS, Koorengevel KM, Steegers EA, Kushner SA (2012). Prevention of postpartum psychosis and mania in women at high risk. Am J Psychiatry.

[CR5] Bonnin CM, Sanchez-Moreno J, Martinez-Aran A, Sole B, Reinares M, Rosa AR (2012). Subthreshold symptoms in bipolar disorder: impact on neurocognition, quality of life and disability. J Affect Disord.

[CR6] Born C, Seitz NN, Grunze H, Vieta E, Dittmann S, Seemuller F (2009). Preliminary results of a fine-grain analysis of mood swings and treatment modalities of bipolar I and II patients using the daily prospective life-chart-methodology. Acta Psychiatr Scand.

[CR7] Courgeau C, Studer R (2018). Do different approaches in population science lead to divergent or convergent models?. Sequence analysis and related apporaches.

[CR8] de Graaf R, ten Have M, van Gool C, van Dorsselaer S (2012). Prevalence of mental disorders and trends from 1996 to 2009. Results from the Netherlands Mental Health Survey and Incidence Study-2. Soc Psychiatry Psychiatr Epidemiol.

[CR9] Denicoff KD, Smith-Jackson EE, Disney ER, Suddath RL, Leverich GS, Post RM (1997). Preliminary evidence of the reliability and validity of the prospective life-chart methodology (LCM-p). J Psychiatr Res.

[CR10] Denicoff KD, Leverich GS, Nolen WA, Rush AJ, McElroy SL, Keck PE (2000). Validation of the prospective NIMH-Life-Chart Method (NIMH-LCM-p) for longitudinal assessment of bipolar illness. Psychol Med.

[CR11] Draisma S, van Zaane J, Smit JH (2015). Data quality indicators for daily life chart methodology: prospective self-ratings of bipolar disorder and alcohol use. BMC Res Notes.

[CR12] Ferrari AJ, Stockings E, Khoo JP, Erskine HE, Degenhardt L, Vos T (2016). The prevalence and burden of bipolar disorder: findings from the Global Burden of Disease Study 2013. Bipolar Disord.

[CR13] Freeman MP, Smith KW, Freeman SA, McElroy SL, Kmetz GE, Wright R (2002). The impact of reproductive events on the course of bipolar disorder in women. J Clin Psychiatry.

[CR14] Gabadinho A, Ritschard G, Müller NS, Studer M (2011). Analyzing and visualizing state sequences in R with TraMineR. J Stat Softw.

[CR15] Geddes JR, Miklowitz DJ (2013). Treatment of bipolar disorder. Lancet.

[CR16] Goodwin FK, Jamison KR (2007). Manic Depressive Illness: Bipolar disorders and Recurrent Depression.

[CR17] Grof P, Robbins W, Alda M, Berghoefer A, Vojtechovsky M, Nilsson A (2000). Protective effect of pregnancy in women with lithium-responsive bipolar disorder. J Affect Disord.

[CR18] Kishi T, Matsuda Y, Sakuma K, Okuya M, Mishima K, Iwata N (2020). Recurrence rates in stable bipolar disorder patients after drug discontinuation v. drug maintenance: a systematic review and meta-analysis. Psychol Med.

[CR19] Kupka RW, Altshuler LL, Nolen WA, Suppes T, Luckenbaugh DA, Leverich GS (2007). Three times more days depressed than manic or hypomanic in both bipolar I and bipolar II disorder. Bipolar Disord.

[CR20] Larsen ER, Saric K (2017). Pregnancy and bipolar disorder: the risk of recurrence when discontinuing treatment with mood stabilisers: a systematic review. Acta Neuropsychiatr.

[CR21] Leverich GS, Nolen WA, Rush AJ, McElroy SL, Keck PE, Denicoff KD (2001). The Stanley Foundation Bipolar Treatment Outcome Network. I Longitudinal Methodology J Affect Disord.

[CR22] McNeil TF, Kaij L, Malmquist-Larsson A (1984). Women with nonorganic psychosis: factors associated with pregnancy's effect on mental health. Acta Psychiatr Scand.

[CR23] Merikangas KR, Jin R, He JP, Kessler RC, Lee S, Sampson NA (2011). Prevalence and correlates of bipolar spectrum disorder in the world mental health survey initiative. Arch Gen Psychiatry.

[CR24] Munk-Olsen T, Laursen TM, Pedersen CB, Mors O, Mortensen PB (2006). New parents and mental disorders: a population-based register study. JAMA.

[CR25] Munk-Olsen T, Laursen TM, Mendelson T, Pedersen CB, Mors O, Mortensen PB (2009). Risks and predictors of readmission for a mental disorder during the postpartum period. Arch Gen Psychiatry.

[CR26] Nolen WA, Luckenbaugh DA, Altshuler LL, Suppes T, McElroy SL, Frye MA (2004). Correlates of 1-year prospective outcome in bipolar disorder: results from the Stanley Foundation Bipolar Network. Am J Psychiatry.

[CR27] Pacchiarotti I, Bond DJ, Baldessarini RJ, Nolen WA, Grunze H, Licht RW (2013). The International Society for Bipolar Disorders (ISBD) task force report on antidepressant use in bipolar disorders. Am J Psychiatry.

[CR28] Parker G, McCraw S, Tavella G, Hadzi-Pavlovic D (2018). Measuring the consequences of a bipolar or unipolar mood disorder and the immediate and ongoing impacts. Psychiatry Res.

[CR29] Post RM, Nolen WA, Kupka RW, Denicoff KD, Leverich GS, Keck PE (2001). The Stanley Foundation Bipolar Network. I. Rationale and methods. Br J Psychiatry Suppl.

[CR30] Post RM, Denicoff KD, Leverich GS, Altshuler LL, Frye MA, Suppes TM (2003). Morbidity in 258 bipolar outpatients followed for 1 year with daily prospective ratings on the NIMH life chart method. J Clin Psychiatry.

[CR31] Roux J, Grimaud O, Leray E (2019). Use of state sequence analysis for care pathway analysis: The example of multiple sclerosis. Stat Methods Med Res.

[CR32] Salim M, Sharma V, Anderson KK (2018). Recurrence of bipolar disorder during pregnancy: a systematic review. Arch Womens Ment Health.

[CR33] Sharma V, Persad E (1995). Effect of pregnancy on three patients with bipolar disorder. Ann Clin Psychiatry.

[CR34] Sharma V, Pope CJ (2012). Pregnancy and bipolar disorder: a systematic review. J Clin Psychiatry.

[CR35] Stevens AWMM, Goossens PJJ, Hoogendoorn AW, Knoppert-vanderKlein EAM, Honig A (2014). The effect of sleep disturbance during pregnancy and perinatal period on postpartum psychopathology in women with bipolar disorder. J Women's Health Care..

[CR36] Stevens A, Goossens PJJ, Knoppert-vander Klein EAM, Draisma S, Honig A, Kupka RW (2019). Risk of recurrence of mood disorders during pregnancy and the impact of medication: A systematic review. J Affect Disord.

[CR37] Suppes T, Leverich GS, Keck PE, Nolen WA, Denicoff KD, Altshuler LL (2001). The Stanley Foundation Bipolar Treatment Outcome Network. II. Demographics and illness characteristics of the first 261 patients. J Affect Disord.

[CR38] Thomson M, Sharma V (2018). Weighing the Risks: the Management of Bipolar Disorder During Pregnancy. Curr Psychiatry Rep.

[CR39] Viguera AC, Cohen LS, Bouffard S, Whitfield TH, Baldessarini RJ (2002). Reproductive decisions by women with bipolar disorder after prepregnancy psychiatric consultation. Am J Psychiatry.

[CR40] Viguera AC, Whitfield T, Baldessarini RJ, Newport DJ, Stowe Z, Reminick A (2007). Risk of recurrence in women with bipolar disorder during pregnancy: prospective study of mood stabilizer discontinuation. Am J Psychiatry.

[CR41] Viguera AC, Tondo L, Koukopoulos AE, Reginaldi D, Lepri B, Baldessarini RJ (2011). Episodes of mood disorders in 2,252 pregnancies and postpartum periods. Am J Psychiatry.

[CR42] Wesseloo R, Kamperman AM, Munk-Olsen T, Pop VJ, Kushner SA, Bergink V (2016). Risk of Postpartum Relapse in Bipolar Disorder and Postpartum Psychosis: A Systematic Review and Meta-Analysis. Am J Psychiatry.

[CR43] Yatham LN, Kennedy SH, Parikh SV, Schaffer A, Bond DJ, Frey BN (2018). Canadian Network for Mood and Anxiety Treatments (CANMAT) and International Society for Bipolar Disorders (ISBD) 2018 guidelines for the management of patients with bipolar disorder. Bipolar Disord.

